# The Relationship of Nutritional Treatments Applied to Patients in a Nutritional Clinic and Mortality

**DOI:** 10.7759/cureus.21579

**Published:** 2022-01-25

**Authors:** Guler Eraslan Doganay, Gulay Ulger

**Affiliations:** 1 Anesthesiology and Reanimation Clinic, Health Sciences University Ankara Atatürk Chest Diseases and Thoracic Surgery Training and Research Hospital, Ankara, TUR

**Keywords:** nutrition, nrs-2002, mortality, nutritional support treatment, malnutrition

## Abstract

Background

Malnutrition is a change in body composition as a result of inadequate nutrient intake or malabsorption. It has a significant effect on morbidity and mortality as a result of increased catabolism in acute and/or chronic diseases of many systems or organs. This study was conducted in a chest diseases branch hospital; applicants to the nutritional clinic are mostly patients with acute or chronic respiratory failure. This study aimed to evaluate the nutritional status of patients at the time of admission to the nutritional clinic and the relationship between nutritional support treatment and mortality.

Materials and methods

The data of 750 patients who applied to the nutritional clinic and consulted clinics, services, and intensive care units were retrospectively analyzed. The patients' demographic data, diagnoses, body mass indexes (BMI), Nutritional Risk Screening (NRS-2002) scores were determined to evaluate malnutrition risks, nutritional support treatments were recorded as enteral, total parenteral, oral enteral supplementation, and nutritional follow-up was initiated. The patients’ main diagnoses were the cause of malnutrition, which were divided into five groups: tuberculosis, chronic obstructive pulmonary disease (COPD), malignancy, neurological diseases, and interstitial lung disease. Thirty-dayand 90-day mortality data were recorded.

Results

A total of 737 patients were included in the study. Of them, 478 (64.8%) were in the geriatric age group. There were 662 (89.9%) patients with an NRS score of ≥3 who were evaluated as malnourished. Enteral nutrition is higher in patients with neurological disease and interstitial lung disease as compared to other diseases. Oral enteral supplementation (OES) is lower in patients with neurological disease and interstitial lung disease compared to other diseases. The rate of nutritional follow-up is higher in patients with interstitial lung disease than in other diseases. The ages and NRS scores of those with mortality were statistically significantly higher than those without mortality. According to the main diagnoses, the rate of COPD patients is significantly lower and the rate of malignant patients significantly higher in patients. The increase in BMI and NRS-2002 score of 3 and above were risk factors for 30-day mortality. OES was the most recommended nutritional product in patients with or without 30-day and 90-day mortality.

Conclusion

Eighty-nine point nine percent (89.9%) of the patients were evaluated as malnourished, and OES was the most recommended nutritional supplement in all patient groups. Mortality was higher in the malignant group and lower in the COPD group as compared to others. There was no correlation between the nutritional product and mortality.

## Introduction

Malnutrition is a change in body composition as a result of inadequate nutrient intake or malabsorption. It appears clinically as the deterioration of physical and mental function due to the decrease in fat-free mass (FFM) and body cell mass (BCM). Nutrition plays an important role in life, especially for patients’ health. Malnutrition has a significant effect on morbidity and mortality as a result of increased catabolism in acute and/or chronic diseases of many systems or organs. However, nutrition is also an important determinant of future health such as cardiovascular disease, diabetes, cancer, and cognitive disease [1]. Therefore, many nutritional screening and evaluation techniques are in clinical use.

The prevalence of malnutrition is high in the population at risk; hence, nutritional assessment should be routinely performed [2]. The high prevalence of malnutrition in the population at risk may increase the risk of developing complications and even decrease the positive effects of treatments [3]. Malnutrition accelerates the catabolic process of diseases. It causes deterioration of physical and mental functions. The catabolic process created by inflammation also deepens malnutrition.

The data obtained from the literature show that Cancer Related Malnutrition (CRM) is still an underrecognized and undertreated problem in clinical practice [4].

Systemic inflammation with inadequate calorie intake, metabolic disorders, depression, fatigue, chemotherapy-induced toxicity, loss of skeletal muscle mass, and changes in acute phase proteins (such as C-reactive protein, albumin, and white blood cell count) contribute to the development of malnutrition in malignant patients [5].

Malnutrition is also frequently observed in chronic obstructive pulmonary disease (COPD). The main reason for this situation is that the increased energy requirement, due to hypermetabolism and increased respiratory work in COPD, cannot be satisfied with impaired nutrition as a result of respiratory difficulties [6].

Malnutrition in COPD leads to a decrease in protein synthesis, independent of respiratory functions, resulting in a decrease in body fat and muscle mass. By creating dysfunction in striated muscles, such as the diaphragm and other accessory respiratory muscles, malnutrition limits exercise capacity and may lead to emphysematous changes in the parenchyma [7].

This study was conducted in a chest diseases branch hospital. Applicants to the nutrition outpatient clinic are mostly from the patient group with acute or chronic respiratory failure. Our malnutrition rates are higher due to the frequently encountered nutritional problems in patients with respiratory tract diseases and malignancy. This study aimed to evaluate the nutritional status at the time of admission and the relationship between nutritional support treatment and mortality in outpatients who applied to the Nutrition Polyclinic in 2019 and were consulted in the service/intensive care unit.

## Materials and methods

The study was designed retrospectively and initiated after approval by the Medical Education Committee of Ataturk Chest Diseases and Thoracic Surgery Education and Research Hospital (approval date and number: 04/03/2021, 716). The data of a total of 750 patients who applied to the nutrition outpatient clinic and were consulted from the outpatient clinic, service, and intensive care unit in 2019 were retrospectively analyzed. The patients' demographic data, diagnoses, body mass indexes (BMI), Nutritional Risk Screening (NRS-2002) scores to evaluate malnutrition risks, and nutritional support treatments initiated for patients with malnutrition risk were recorded. Bodyweight and height at the time of application were recorded in line with the statement of the patient or family members. The nutritional product recommended to the patient was grouped as enteral, total parenteral, oral enteral supplementation, and nutritional follow-up. Thirteen patients with nutritional deficiency and patients with no risk were excluded from the study.

Nutritional risk assessment was performed within the 48 hours of hospitalization using the NRS-2002 as recommended by the European Society for Clinical Nutrition and Metabolism (ESPEN) [8]. The NRS includes assessment of the patient's nutritional status (based on weight loss, body mass index (BMI), and food intake) and disease severity. Each parameter is scored between 0 and 3, and patients get extra points if they are 70 years or older.

Initiated nutritional support

Total parenteral nutrition (TPN): Contains an amino acid solution for infusion, glucose solution, and lipid emulsion. The nutritional product in the readymade drug formulation was defined as an intravenous (peripheral or central) infusion to provide >10 kcal/kg/day energy for at least three days.

Enteral nutrition (EN): Was defined as the continued use of commercial formulas providing >10 kcal/kg/day of energy via nasogastric or percutaneous endoscopic gastrostomy (PEG) for at least three days.

Oral enteral supplementation: Was defined as the continuous use of commercial formulas taken orally for at least three days, providing >10 kcal/kg/day of energy.

Nutritional follow-up: Was recommended to patients with a normal malnutrition screening score or those who did not start a nutritional product.

The patients’ main diagnoses that are the cause of malnutrition were divided into five groups: tuberculosis, COPD, malignancy, neurological diseases, and interstitial lung disease. The patients or their relatives were called from the contact phone numbers in the hospital system and 30-day and 90-day mortality data were recorded.

Statistical analyses

Data analyses were performed using SPSS for Windows, version 22.0 (IBM Corp., Armonk, NY). Whether the distribution of continuous variables was normal or not was determined by the Kolmogorov Smirnov test and kurtosis and skewness values. The Levene test was used for the evaluation of homogeneity of variances. Continuous data were described as mean ± standard deviation (SD) and minimum and maximum. Categorical data were described as a number of cases (%). Statistical analysis differences in normally distributed variables between two independent groups were compared using the student’s t-test. Categorical variables were compared using Pearson’s chi-square test or Fisher’s exact test. Univariate and multivariate logistic regression analyses were performed to assess the association between 30-day and 90-day mortality and the risk factors findings. A p-value of < 0.05 was accepted as a significant level on all statistical analyses.

## Results

The number of applications to the nutrition outpatient clinic was 983 in 2019. Of these patients, 246 were excluded due to missing data. Seven-hundred thirty-seven (737) patients were included in the study. Four-hundred seventy-eight (478; 64.8%) of these patients were in the geriatric age group.

The average age was 68.77 ± 14.11 years, and 535 (72.6%) of them were men. Their mean BMI was 22.72 ± 4.90 kg/m^2^. There were 662 (89.9%) patients with a mean NRS score of ≥3 who were evaluated as malnourished (Table 1).

**Table 1:** Characteristics of all patients applied to the nutrition outpatient Continuous variables are expressed as either mean ± standard deviation (SD) or minimum-maximum value and categorical variables are expressed as either frequency (percentage). BMI: Body Mass Index; NRS: Nutritional Risk Screening; COPD: Chronic Obstructive Pulmonary Disease; TPN: Total Parenteral Nutrition; OES: Oral Enteral Supplementation

		Patients (n:737)
Age, ±SD (min-max)		68.77 ± 14.11 (18-100)
Gender	Male	535 (72.6%)
	Female	202 (27.4%)
BMI, ±SD (min-max)		22.72 ± 4.90 (10.41-42.58)
NRS-2002 score	±SD (min-max)	3.50 ± 1.09 (1-7)
	<3	74 (10.1%)
	≥3	662 (89.9%)
Main diagnoses	COPD	407 (55.2%)
	Malignancy	175 (23.7%)
	Neurological diseases	105 (14.2%)
	Tuberculosis	27 (3.7%)
	Interstitial lung disease	23 (3.1%)
Nutritional follow-up	-	723 (98.1%)
	+	14 (1.9%)
TPN	-	659 (89.4%)
	+	78 (10.6%)
Enteral nutrition	-	610 (82.8%)
	+	127 (17.2%)
OES	-	219 (29.7%)
	+	518 (70.3%)
30-day mortality	-	427 (57.9%)
	+	310 (42.1%)
90-day mortality	-	319 (43.3%)
	+	418 (56.7%)

When the patients are grouped according to their diagnosis, 407 patients (55.2%) have COPD, 175 patients (23.7%) have a diagnosis of malignancy, 105 patients (14.2%) have a diagnosis of neurological disease, 27 patients (3.7%) have tuberculosis, 23 patients (3.1%) were diagnosed with interstitial lung disease. Three-hundred ten (42.1%) patients died within 30 days, and 418 (56.7%) patients died within 90 days (Table 1).

While there is no significant difference in terms of TPN according to co-morbidities, there is a significant difference in terms of enteral (p < 0.001), OES (p < 0.001), and nutritional follow-up (p = 0.09). Enteral nutrition is higher in patients with neurological disease and interstitial lung disease compared to other diseases. OES is lower in patients with neurological disease and interstitial lung disease compared to other diseases. The rate of nutritional follow-up is higher in patients with interstitial lung disease than in other diseases (Table 2).

**Table 2:** Recommended nutritional support according to the main diagnosis Categorical variables are expressed as either frequency or percentage. Categorical variables were compared using Pearson’s chi-square test or Fisher s exact test. Statistically significant p-values are in bold. COPD: Chronic Obstructive Pulmonary Disease TPN: Total Parenteral Nutrition OES: Oral Enteral Supplementation

		Main Diagnoses										
		COPD		Malignancy		Neurological Diseases		Tuberculosis		Interstitial Lung Disease		p
		n	(%)	n	(%)	n	(%)	n	(%)	n	(%)	
TPN	-	364	(89.4%)	162	(92.6%)	91	(86.7%)	22	(81.5%)	20	(87.0%)	0.328
	+	43	(10.6%)	13	(7.4%)	14	(13.3%)	5	(18.5%)	3	(13.0%)	
Enteral	-	365	(89.7%)	150	(85.7%)	55	(52.4%)	23	(85.2%)	17	(73.9%)	<0.001
	+	42	(10.3%)	25	(14.3%)	50	(47.6%)	4	(14.8%)	6	(26.1%)	
OES	-	95	(23.3%)	39	(22.3%)	65	(61.9%)	9	(33.3%)	11	(47.8%)	<0.001
	+	312	(76.7%)	136	(77.7%)	40	(38.1%)	18	(66.7%)	12	(52.2%)	
Nutritional follow-up	-	397	(97.5%)	174	(99.4%)	105	(100.0%)	27	(100.0%)	20	(87.0%)	0.009
	+	10	(2.5%)	1	(0.6%)	-		-		3	(13.0%)	

It was examined whether there was a difference in terms of related variables between 30-day mortality and those who did not. According to the results, the ages of those with mortality were statistically significantly higher than those without mortality (p = 0.047). NRS-2002 scores of those with mortality were statistically significantly higher than those without mortality (p < 0.001). In addition, the rate of those with an NRS-2002 score of 3 and above is statistically significantly higher than those who did not (p = 0.006). In the main diagnoses, the rate of COPD patients with mortality is statistically significantly lower than those without (p < 0.001). The rate of malignant patients in patients with mortality is statistically significantly higher than those without (p < 0.001) (Table 3).

**Table 3:** Comparison of patients with and without 30-day mortality in terms of risk factors Continuous variables are expressed as either the mean ± standard deviation (SD) and categorical variables are expressed as either frequency or percentage. Continuous variables were compared with the Student t-test and categorical variables were compared using Pearson’s chi-square test or Fisher’s exact test. Statistically significant p-values are in bold. BMI: Body Mass Index; NRS: Nutritional Risk Screening; COPD: Chronic Obstructive Pulmonary Disease; TPN: Total Parenteral Nutrition; OES: Oral Enteral Supplementation

	30-day mortality (+) (n:310)	30-days mortality (-) (n:420)	p
Age, ±SD	69.98 ± 13.34	67.89 ± 14.60	0.047
Gender			0.742
Male	227 (73.2%)	308 (72.1%)	
Female	83 (26.8%)	119 (27.9%)	
BMI, ±SD	23.04 ± 4.88	22.49 ± 4.90	0.126
NRS-2002 score, ±SD	3.66 ± 1.11	3.38 ± 1.06	<0.001
<3	20 (6.5%)	54 (12.7%)	0.006
≥3	290 (93.5%)	372 (87.3%)	
Main Diagnoses			
COPD	142 (45.8%)	265 (62.1%)	<0.001
Malignancy	98 (31.6%)	77 (18.0%)	<0.001
Neurological diseases	48 (15.5%)	57 (13.3%)	0.413
Tuberculosis	13 (4.2%)	14 (3.3%)	0.514
Interstitial lung disease	9 (2.9%)	14 (3.3%)	0.772
Nutritional follow-up			0.114
-	307 (99.0%)	416 (97.4%)	
+	3 (1.0%)	11 (2.6%)	
Total Parenteral (TPN)			0.496
-	280 (90.3%)	379 (88.8%)	
+	30 (9.7%)	48 (11.2%)	
Enteral			0.090
-	248 (80.0%)	362 (84.8%)	
+	62 (20.0%)	65 (15.2%)	
Oral Enteral Supplementation (OES)			0.638
-	95 (30.6%)	124 (29.0%)	
+	215 (69.4%)	303 (71.0%)	

It was examined whether there was a difference in terms of related variables between patients with and without 90-day mortality. According to the results, the ages of patients with mortality were statistically significantly higher than patients without mortality (p = 0.003). NRS-2002 scores of patients with mortality were statistically significantly higher than patients without mortality (p = 0.001). In addition, the rate of patients with mortality NRS-2002 score of 3 and above is statistically significantly higher than patients without mortality (p = 0.003). The rate of COPD patients with mortality is statistically significantly lower than those without mortality (p < 0.001). The rate of malignancy in patients with mortality is statistically significantly higher than in patients without mortality (Table 4).

**Table 4:** Comparison of Patients With and Without 90-Day Mortality in terms of Risk Factors Continuous variables are expressed as either the mean ± standard deviation (SD) and categorical variables are expressed as either frequency or percentage. Continuous variables were compared with the student's t-test and categorical variables were compared using Pearson’s chi-square test or Fisher’s exact test. Statistically significant p-values are in bold. BMI: Body Mass Index; NRS: Nutritional Risk Screening; COPD: Chronic Obstructive Pulmonary Disease; TPN: Total Parenteral Nutrition; OES: Oral Enteral Supplementation

	90-day mortality (+) (n:418)	90-day mortality (-) (n:319)	p
Age, ±SD	70.12 ± 13.24	67.00 ± 15.02	0.003
Gender			0.794
Male	305 (73.0%)	230 (72.1%)	
Female	113 (27.0%)	89 (27.9%)	
BMI, ±SD	23.01 ± 4.69	22.34 ± 5.34	0.068
NRS-2002 score, ±SD	3.61 ± 1.09	3.35 ± 1.07	0.001
<3	30 (7.2%)	44 (13.8%)	0.003
≥3	387 (92.8%)	275 (86.2%)	
Main Diagnoses			
COPD	198 (47.4%)	209(65.5%)	<0.001
Malignancy	130 (31.1%)	45 (14.1%)	<0.001
Neurological diseases	65 (15.6%)	40 (12.5%)	0.247
Tuberculosis	16 (3.8%)	11 (3.4%)	0.786
Interstitial lung disease	9 (2.2%)	14 (4.4%)	0.084
Nutritional follow-up			0.007
-	415 (99.3%)	308 (96.6%)	
+	3 (0.7%)	11 (3.4%)	
Total Parenteral (TPN)			0.363
-	370 (88.5%)	289 (90.6%)	
+	48 (11.5%)	30 (9.4%)	
Enteral			0.434
-	342 (81.8%)	268 (84.0%)	
+	76 (18.2%)	51 (16.0%)	
Oral Enteral Supplementation (OES)			0.650
-	127 (30.4%)	92 (28.8%)	
+	291 (69.6%)	227 (71.2%)	

In order to determine the factors affecting 30-day mortality, univariate logistic regression analysis was applied first. Variables with p < 0.25 in the univariate logistic regression analysis were included in the multivariate logistic regression analyses. The variable logistic regression analysis model used factors with a p-value < 0.05 and affect 30-day mortality. Considering the results, an increase in BMI and NRS-2002 score of 3 and above are risk factors for 30-day mortality. COPD disease appears to be less risky for 30-day mortality. Malignancy is a risk factor for 30-day mortality (Table 5).

**Table 5:** Results of univariate and multivariate logistic regression analysis applied to determine risk factors affecting 30-day mortality Wald: test statistics. OR: odds ratio. Statistically significant p-values are in bold. BMI: Body Mass Index; NRS: Nutritional Risk Screening; COPD: Chronic Obstructive Pulmonary Disease; TPN: Total Parenteral Nutrition; OES: Oral Enteral Supplementation

30-Day Mortality	Univariate Logistic Regression				Multivariate Logistic Regression				
	Wald	p	OR	95% C.I.for OR	Wald	p	OR	95% C.I.for OR	
Age	3.927	0.048	1.011	(1.000-1.022)	2.596	0.107	1.009	(0.998-1.021)	
Gender (ref:female)	0.108	0.742	1.057	(0.761-1.468)					
BMI	2.338	0.126	1.024	(0.993-1.055)	6.774	0.009	1.044	(1.011-1.078)	
NRS-2002 (ref:<3)	7.419	0.006	2.105	(1.232-3.596)	5.598	0.018	1.985	(1.125-3.504)	
COPD	19.003	<0.001	0.517	(0.384-0.695)	4.060	0.044	0.660	(0.441-0.989)	
Malignancy	17.920	<0.001	2.101	(1.490-2.963)	6.506	0.011	1.817	(1.148-2.876)	
Neurological diseases	0.669	0.413	1.189	(0.785-1.801)					
Tuberculosis	0.424	0.515	1.291	(0.598-2.787)					
Interstitial lung disease	0.084	0.772	0.882	(0.377-2.065)					
Nutritional follow-up	2.305	0.129	0.370	(0.102-1.336)	0.511	0.475	1.631	(0.160-2.343)	
TPN	0.463	0.496	0.846	(0.523-1.369)					
Enteral	2.859	0.091	1.392	(0.949-2.043)	1.659	0.198	1.316	(0.866-2.000)	
OES	0.222	0.638	0.926	(0.673-1.275)					

**Figure 1:**
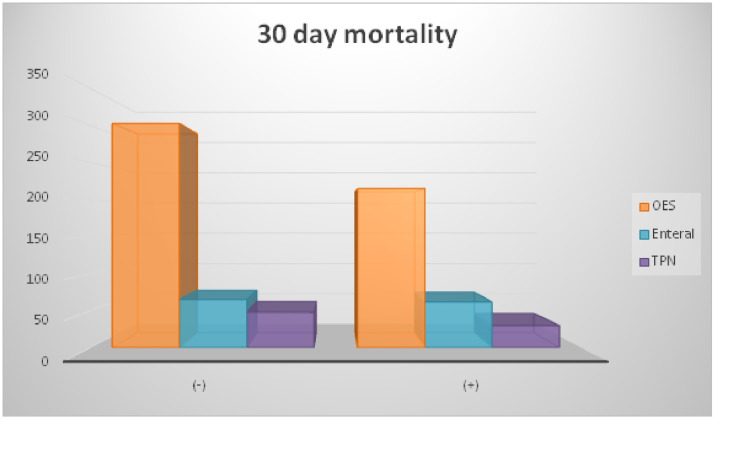
Distribution of nutritional support given in patients with and without 30-day mortality TPN: Total Parenteral Nutrition; OES: Oral Enteral Supplementation

OES was the most recommended nutritional product in patients with or without 30-day mortality. Afterward, enteral and total parenteral nutrition was given, respectively, and they were not associated with 30-day mortality (Figure 1).

In order to determine the factors affecting 90-day mortality, univariate logistic regression analysis was applied first. Variables with a p-value of < 0.25 in the univariate logistic regression analysis were included in the multivariate logistic regression analysis. The variable logistic regression analysis model used factors with a p-value of < 0.05 and affect 90-day mortality. Considering the results, age, and increase in BMI and NRS-2002 score of 3 and above are risk factors for 90-day mortality (Table 6).

**Table 6:** Results of univariate and multivariate logistic regression analysis applied to determine risk factors affecting 90-day mortality Wald: test statistics. OR: odds ratio. Statistically significant p-values are in bold. BMI: Body Mass Index; NRS: Nutritional Risk Screening; COPD: Chronic Obstructive Pulmonary Disease; TPN: Total Parenteral Nutrition; OES: Oral Enteral Supplementation

90-Day Mortality	Univariate Logistic Regression				Multivariate Logistic Regression				
	Wald	p	OR	95% C.I.for OR	Wald	p	OR	95% C.I.for OR	
Age	8.687	0.003	1.016	(1.005-1.027)	4.718	0.030	1.014	(1.001-1.026)	
Gender (ref:female)	0.068	0.794	1.044	(0.754-1.447)					
BMI	3.323	0.068	1.028	(0.998-1.060)	8.274	0.004	1.049	(1.015-1.084)	
NRS-2002 (ref:<3)	8.431	0.004	2.064	(1.266-3.366)	5.610	0.018	1.908	(1.118-3.258)	
COPD	23.787	<0.001	0.474	(0.351-0.640)	1.906	0.167	0.564	(0.250-1.272)	
Malignancy	27.600	<0.001	2.748	(1.885-4.008)	2.770	0.096	2.066	(0.879-4.855)	
Neurological diseases	1.338	0.247	1.284	(0.840-1.963)	0.098	0.755	0.868	(0.358-2.106)	
Tuberculosis	0.074	0.786	1.114	(0.510-2.436)					
Interstitial lung disease	2.871	0.090	0.479	(0.205-1.122)	0.204	0.652	0.756	(0.225-2.545)	
Nutritional follow-up	5.936	0.015	0.202	(0.056-0.732)	2.512	0.113	0.336	(0.087-1.294)	
TPN	0.824	0.364	1.520	(0.772-2.023)					
Enteral	0.610	0.435	1.168	(0.791-1.723)					
OES	0.206	0.650	0.929	(0.675-1.278)					

**Figure 2:**
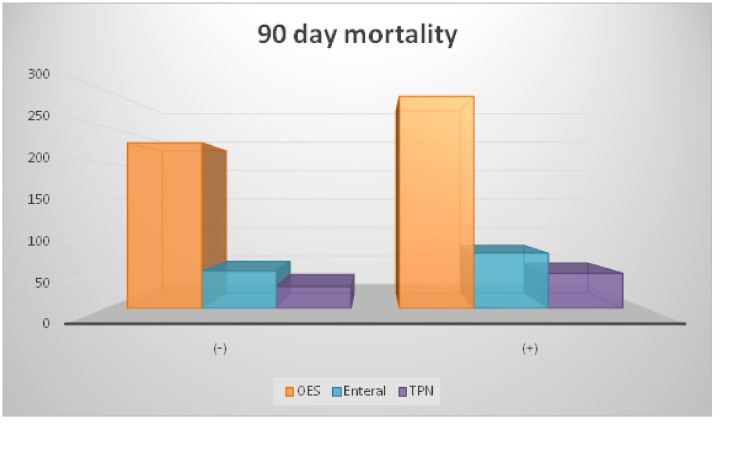
Distribution of nutritional support given in patients with and without 90-day mortality TPN: Total Parenteral Nutrition; OES: Oral Enteral Supplementation

OES was the most recommended nutritional product in patients with or without 90-day mortality. Afterward, enteral and total parenteral nutrition was given, respectively, and they were not associated with 90-day mortality (Figure 2).

## Discussion

Disease-related malnutrition is still a major problem today. It is important to evaluate the nutritional status of patients during hospitalization and to plan nutritional intervention. Malnutrition affects the prognosis in patients with chronic diseases as well as in malignant patients. Nutritional support and prognosis-mortality studies are limited in the literature.

Our study with all patients (outpatient clinic/service/intensive care unit) for whom support was initiated in the nutrition outpatient clinic showed that, in all patients in whom nutritional support was started, the most frequently used supplement in each disease group was OES. Among the patients who received nutritional support, the COPD group was less mortal while the mortality was higher in the malignant group. Age and NRS-2002 scores were higher in patients who received nutritional support compared to those who did not develop mortality. No relationship was found between the nutritional product started and mortality.

There are studies about the relationship between BMI and nutritional status with mortality [9-12]. In our study, an increase in BMI and NRS-2002 score above 3 were risk factors for 30-day and 90-day mortality while advanced age was found to be a risk factor for 90-day mortality.

NRS-2002 is an accepted indicator that determines the risk of developing malnutrition for patients in the hospital [13]. In a study conducted in the intensive care unit, NRS 2002 was used as a screening test and 99.4% of patients were considered malnourished with NRS-2002 > 3 [14]. In our study, 89.9% of the patients were found to be malnourished.

In the study of Mingwei Zhu et al., the nutritional risk and prevalence of malnutrition at discharge were found to be higher than at admission, and the clinical outcomes of all patients with nutritional risk were evaluated as worse [15]. In our study, 98.1% of the patients who applied to the nutrition outpatient clinic were given nutritional support, and only 1.9% of the patients were evaluated as adequate and nutritional follow-up was performed. Nutritional monitoring is valuable in recognizing the deficiency.

Waitzberg et al. emphasized in their study that, like our study, malnutrition increases the incidence of mortality [2]. Weight loss occurs in about half of COPD patients. Both fat and lean body mass decrease [16]. Malnutrition can be seen in COPD patients even if there is no weight loss. Malnutrition leads to atrophy in respiratory muscles and thus weakening of respiratory muscles, increasing the risk of pneumonia due to impaired immunity [17]. Since COPD patients receive long hours of non-invasive mechanical ventilation therapy, which also causes decreases in their oral intake, high-calorie support provides more effective nutrition in the COPD patient group. It was shown that there is a relationship between the nutritional status of COPD patients and their mechanical ventilation needs, mortality, and morbidity [18].

It was stated in a study that malignancy was an independent risk factor for malnutrition, and it was recommended to evaluate the nutritional status at the time of hospitalization in hospitalized patients [19]. Nutritional deficiency develops especially during and after surgery or radiotherapy/chemotherapy due to taste disturbance and loss of appetite in patients with malignancy, therefore, it was recommended to add OES to the diet in a study [20].In our study, the most recommended nutritional support in malignant patients was OES. OES are ready-to-drink solutions developed to provide energy and nutrient density. They are products with well-established clinical effects and optimal cost-effectiveness [21-23]. Although nutritional support was started in malignant patients in our study, the 30-day and 90-day mortality rates were found to be higher than in the COPD patient group. This can be explained by the faster course of the catabolic process in malignant patients.

Nutrition plays a very important role in the prevention, delay, and treatment of diseases related to aging. In addition, pharmacokinetic and pharmacodynamic changes may occur with aging [24-25]. Besides, malnutrition has been reported to be associated with poor prognosis, especially in elderly patients, patients with malignancy, and patients with chronic diseases [26]. The catabolic process is caused by inflammatory diseases with old age, the risk of malnutrition ve mortality increases [27]. Nutrition is of great importance in ensuring adequate cognitive and physical functions in the elderly population, reducing the risk of chronic diseases, and preventing the risk of malnutrition [28]. The main causes of weight loss in elderly patients are inadequate nutritional intake (anorexia and malnutrition of old age) and/or catabolic process (cachexia) and sarcopenia due to chronic diseases. In the study of Sullivan et al., it was emphasized that weight loss increases mortality and morbidity [12].

It is convenient to start with OES for every patient with oral intake, which provides calorie and protein support. However, enteral products are recommended in patients who cannot take oral medications due to neurological dysphagia or malignancy but can achieve enteral absorption and are followed up on mechanical ventilators.

Gastrointestinal obstruction, dysfunctional gastrointestinal tract, prolonged ileus, severe diarrhea or malabsorption, enterocolitis, gastrointestinal hemorrhage, uncontrollable vomiting, high-output (>500 mL/day) fistulas, severe pancreatitis, mesenteric ischemia, or peritonitis are indications for parenteral nutrition.

In a study conducted in the intensive care unit, it was found that enteral nutrition support and TPN treatment were found to be higher in the group of patients who died while those who received oral support were found to be higher in the survivor group [10]. Conversely, mortality rates were high in patients who received OES in our study. Because our study includes both outpatients and inpatients.

The limitations of the study were single-center and retrospective. Since the study was conducted in a chest diseases branch hospital, the number of postoperative patients, burns, trauma, non-pulmonary malignancies, and neurological patients could not be equal in the study population.

## Conclusions

Eighty-nine point nine (89.9%) of the patients who applied or consulted the Chest Diseases Hospital Nutrition Polyclinic were evaluated as malnourished, and OES was the most recommended nutritional supplement in all patient groups (COPD, malignancy, tuberculosis, interstitial lung disease, and neurological diseases). Mortality was higher in the malignant patient group who received nutritional support compared to COPD patients. Advanced age and high NRS-2002 score were found to be risk factors for mortality, but no correlation was found between the nutritional product started and mortality. Our study is remarkable in that it indicates the importance and necessity of nutritional support therapy in chest diseases. There is a need for larger studies to provide adequate and early nutritional support in patient populations in special branch hospitals.
